# The Editor's Letter-Box

**Published:** 1893-08-19

**Authors:** J. Brendon Curgenven

**Affiliations:** Teddington Hall


### Editor's Letter-Box.

[Our correspondents are reminded that proxility is a great bar to publication, and that brevity of style and conciseness of statement greatly facilitate early insertion.]

Sir,—From investigations as to the suitability of the various popular disinfectants and other recognised antiseptics, for inunction in infectious diseases, carried on for many years, I can only say now as I did in 1891 in my paper read at the International Congress "that not one is suitable for inunction over the whole surface of the body of a young child; some because they are too poisonous, others because their solutions in water or oil or their admixture with fats. Vaseline or lanolin are injurious by obstructing the natural action of the skin, so necessary during fever, beyond even the requirement of health."

Mr. Kingzett may be a good chemist but he is not a physiologist, otherwise he would know that the resinous "residue" which, he says, "the better qualifies" Sanitas oil for inunction would so obstruct the action of the skin that fatal results would follow. At one time it was the practice in Lincolnshire to anoint horses with olive oil after clipping them, but several died in consequence of pnuemonia and nephritis, and the practice was abandoned. I therefore must decline Mr. Kingzett's challenge to be a party to trying such an experiment as he proposes of anointing children in fever with his "Sanitas" oil which would leave the resinous "residue" on the skin as a coat of varnish.

He must allow that Dr. W. H. Dickinson of St. George's Hospital is an authority on the physiology of the skin and kidneys. In his remarks, from the chair, on my last paper he said: "One point in favour of the method advocated was the complete volatility of the substance employed for inunction, for he held strongly that inunction of a scarlet fever patient with any form of fat or fixed oil was deleterious and especially liable to favour the development of renal complications." (*British Medical Journal*, Feb. 4th, 1893.)

For confirmation of my statements of facts concerning the method of the antiseptic treatment which I advocate, and not of my "hypothetical opinions," allow me to refer Mr. Kingzett to an article with cases by a well known medical man in this month's *Medical Magazine*.—I am, &c., Yours truly,

J. Brendon Curgenven.

Teddington Hall, August 7th, 1893.

